# Integrating social determinants of health principles into the preclinical medical curriculum via student-led pedagogical modalities

**DOI:** 10.1186/s12909-023-04152-0

**Published:** 2023-04-04

**Authors:** Krisandra Kneer, Erik Zhang, Tyler Harkness, Timothy Lahey, Karen M. Lounsbury

**Affiliations:** 1 Class of 2025, Larner College of Medicine, University of Vermont, Burlington, VT 05405 United States of America; 2 Class of 2024, Larner College of Medicine, University of Vermont, Burlington, VT 05405 United States of America; 3 Departments of Medicine/Ethics, Larner College of Medicine, University of Vermont, Burlington, VT 05405 United States of America; 4 Department of Pharmacology/Office of Medical Education, Larner College of Medicine, University of Vermont, Burlington, VT 05405 United States of America

**Keywords:** Social determinants of health, Social medicine curriculum, Social medicine theme of the week, Infographic, Student-driven, Peer teaching, Multi-media resources

## Abstract

**Background:**

Dismantling structural inequities in health care requires that physicians understand the impacts of social determinants of health (SDH). Although many medical schools incorporate SDH education, integration of these principles into the preclinical curriculum remains challenging.

**Methods:**

Students and faculty at the University of Vermont, Larner College of Medicine developed the Social Medicine Theme of the Week (SMTW), a peer-teaching approach to integrating SDH topics across the preclinical curriculum as part of a broader social medicine curriculum. Students created objectives to link SDH-related topics to the weekly curriculum and presented them to the class. Student innovation led to the incorporation of creative online infographics that were published in the curriculum calendar. First year medical students and faculty members were surveyed to assess preferences and educational impact of the SMTW announcements with accompanying infographics.

**Results:**

Of the 40 student respondents, 77.5% reported that their knowledge of SDH had improved due to the SMTW. Most students (82.5%) preferred the infographic modality over traditional teaching modalities. Faculty respondents reported limited engagement with the SMTW and, although they supported the need for these objectives, many (61%) found it difficult to integrate SDH content into their class materials.

**Conclusion:**

Student-led infographics are a popular method of integrating SDH content in the preclinical curriculum that can be optimized through faculty orientation and support. Success for this type of instruction requires opportunities for student developers, integration and formal assessment of objectives, faculty engagement and training, and institutional support for creating and delivering a robust social medicine curriculum.

**Supplementary Information:**

The online version contains supplementary material available at 10.1186/s12909-023-04152-0.

## Background

Physicians must consider social determinants of health (SDH) to provide quality health care to their patients and to work towards dismantling structural health inequities [1]. In combination with medical training experiences, early exposure to SDH concepts has been shown to increase the likelihood of students choosing practice sites in areas of high need [2]. The strategic plan of the American Medical Association (AMA) recognizes that the impact of health disparities within medical education must be prioritized, otherwise the existing structure of inequity will be maintained and propagated by future physicians [3–5].

Meaningful integration of SDH into the curriculum remains a major barrier to successful SDH teaching [6]. A recent AMA consortium with respondents from 29 medical schools found that 36% still consider the teaching of SDH to be a low priority, and opinions still differ regarding whether combating health inequities lies within the realm of physician responsibilities [7–9]. When SDH concepts are included in the curriculum, the topics are often presented as a one-time service-learning trip or small group discussions, and the content is often not associated with a specific learning objective or assessment [10, 11]. Even when taught as a self-contained course, medical schools have not adequately integrated the basic science and societal aspects of medicine together [12]. It is imperative for these topics to be woven into the curriculum longitudinally in a manner that explicitly matches the social medicine and health disparities information to basic science principles.

At the University of Vermont, Larner College of Medicine (LCOM), we have developed an innovative social medicine curriculum that focuses on integrating the SDH material directly into basic science and clinical training [13, 14]. One facet of this social medicine curriculum is the Social Medicine Theme of the Week (SMTW), a student-led modality that directly connects SDH and basic science principles through in-class announcements. Student volunteers share an in-person announcement to introduce and provide context to the theme using objectives that illustrate the connections between social determinants of health and basic science topics. Our previous analysis of the SMTW implementation showed that students valued the student-driven nature of the themes and that the objectives and format are supported by faculty who have expertise and/or strong interest in social justice [13]. To build on those previous findings, this research focused on the student-led use of infographics as an innovative way to link social medicine concepts to the foundational science curriculum.

An infographic is a visual format, typically including charts, diagrams, or summarized statistics, that is useful for conveying information on a given topic. Infographics allow a more effective flow of information compared with traditional teaching methods and have been shown to increase reading comprehension, facilitate data interpretation, and lower cognitive load among learners [15, 16]. The primary educational intervention applied by our team involves weekly infographic-styled online newsletters centering each individual SMTW with links to resources accessible to students through their course calendar. SMTW infographics are easily integrated into virtual learning, which became more important and necessary during the COVID-19 pandemic.

Here we describe the design and implementation of the student-led SMTW infographic and evaluate survey feedback from first year medical students and teaching faculty members.

## Methods

As detailed in our previous work [14], the SMTW currently exists in the context of three core components of a social medicine curriculum: (1) a 3-year longitudinal course called Professionalism, Communication, and Reflection (PCR), consisting of a series of preceptor-led small-group conversations involving interpersonal, social justice, and social medicine topics; (2) a series of ethics sessions across the preclinical curriculum; and (3) integrated, longitudinal social medicine content driven by student-faculty collaboration. The SMTW is a formal component of this third category, as student leaders and volunteers work in conjunction with curriculum and course directors to make improvements in the content and delivery of SMTW topics.

### Design of the social medicine theme of the week

Each SMTW is designed through a collaboration among students, course directors, ethics faculty, and the preclinical level director. The SMTW is comprised of objectives that connect preclinical coursework to social medicine principles, a web-based infographic created by medical students that uses text, images, and external resource links, and a weekly in-person announcement by student volunteers during class time to introduce and provide context to the theme and the infographic.

### Identifying a social medicine theme of the week

Each SMTW is tailored to account for the social determinants of health, public health principles, systemic issues, and advocacy strategies that align to that week’s preclinical curriculum. For example, in a week covering inflammatory bowel disease, bowel obstructions, and colorectal cancers, the SMTW covers racial disparities in colorectal cancers, colorectal cancer screening, treatment, and the underlying systemic reasons for those elements. Because there are many social medicine topics of varying complexity available for discussion and almost all topics are mentioned at most tangentially if at all within the established clinical curriculum, students have the opportunity to bring attention to a near unlimited range of topics provided their personal interests and ability to gather relevant information and perspectives. Themes are screened and edited with the assistance of core organizers maintaining oversight of consistency and balance between topics over the year. An outline of the weekly alignment of the SMTW across the preclinical curriculum and example infographics are shown in **Appendix**A and B, respectively.

### Recruitment of students, infographic and announcement generation

Members of the student Social Justice Coalition recruit student volunteers to work on the SMTW project. Students generate infographics via the web-based infographic generator Venngage.com. The general structure of each infographic includes a block of text providing a summary of the theme, data visualizations relevant to the ideas presented in the text (graphs, timelines, statistics, etc.), and a resource section that provides hyperlinks to materials for self-directed learning. The preclinical level director approves the content and disseminates a website link to each SMTW infographic newsletter via their learning management system. Course directors and student presenters alert faculty speakers in advance of the need to make a 5-minute announcement about the SMTW. Announcements typically include content derived from the infographic material with few formal requirements; the primary objective for the announcements is to direct students to the more detailed infographics and to present opportunities for future discussions about relevant social medicine topics.

### Survey design/evaluative strategy

Online surveys were provided to first year medical students and preclinical faculty members to evaluate the usefulness of the SMTW and its role in SDH education. Free-text prompts enabled respondents to detail positive and negative experiences with the SMTW and its incorporation of social medicine topics into preclinical course curriculum, as well as provide suggestions for the project moving forward. The student survey (**Appendix**C) and the faculty survey (**Appendix**D) included a combination of multiple choice, Likert scale, and free response questions that evaluated attitudes towards SMTW content and objectives, elements of information distribution, and integration into existing curricular structures. Surveys remained open to responses for two weeks. Incomplete surveys were excluded from the analysis. Microsoft Excel was used to carry out simple descriptive statistical analyses and visualization of survey results.

## Results

### Quantitative survey results

#### Students

Forty out of 123 first-year medical students (32.5%) responded to the survey. Detailed results are shown in Table 1. All student respondents were aware of the SMTW, and most students (92.5%) found it helpful for synthesizing information regarding SDH into coursework. Most students reported a “good balance” of race-related topics in the curriculum (55%). Most students reported too little content on sex and gender (57.5%), lesbian, gay, bisexual, trans and queer plus (LGBTQ+) issues (67.5%), global health (55%), and structural violence (62.5%). Most students (72.5%) indicated that their knowledge of SDH had increased during their first year in medical school and that there should be more sessions or learning opportunities to address SDH.

**Fig. 1:**
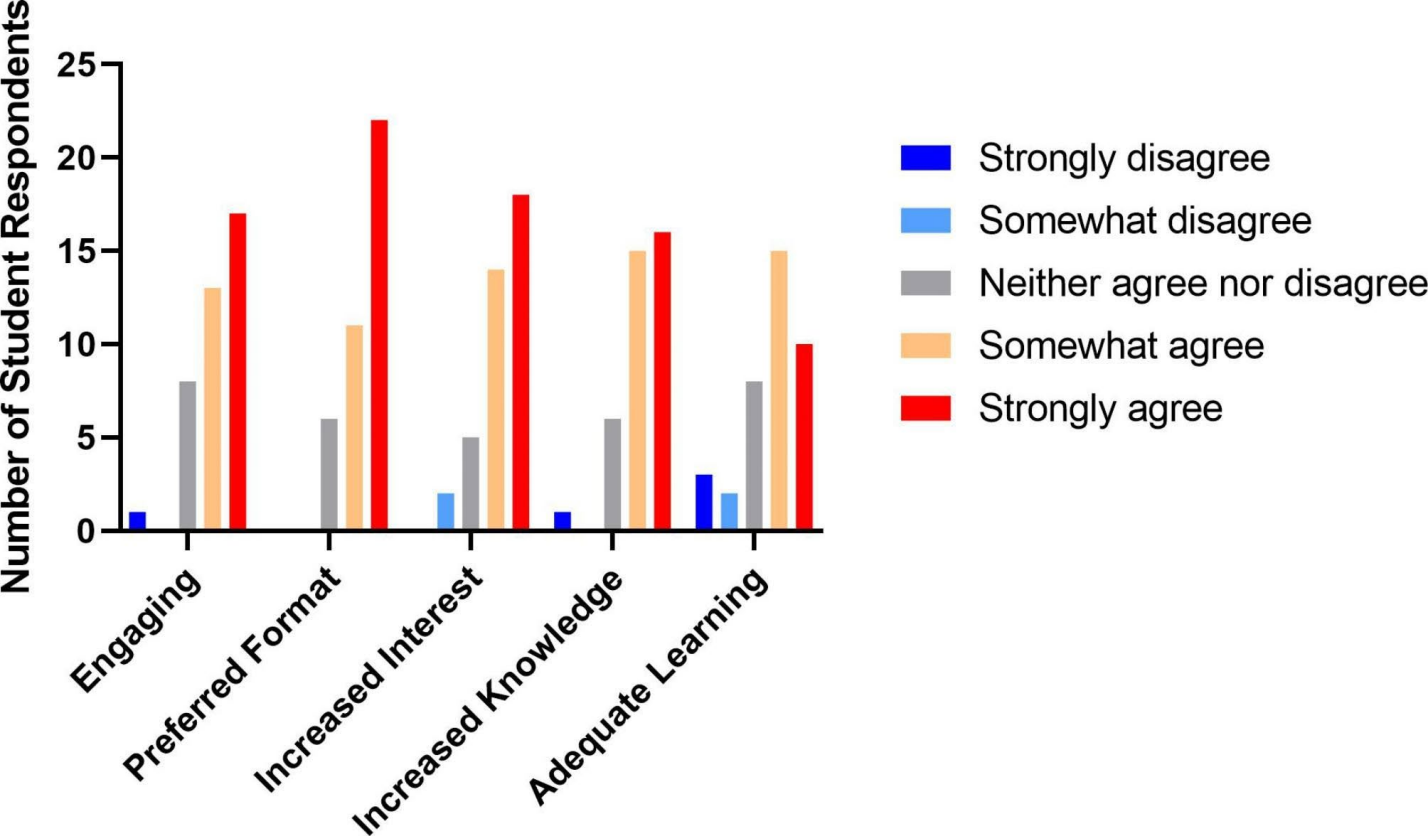
Student Opinions on Social Medicine Theme of the Week Infographics. Students responded to Likert scale questions about whether they believed SMTW infographics were more engaging, provided a better format, increased their interest in SDH, increased their knowledge of SDH, and provided adequate learning compared to traditional formats. (n = 40)

**Fig. 2:**
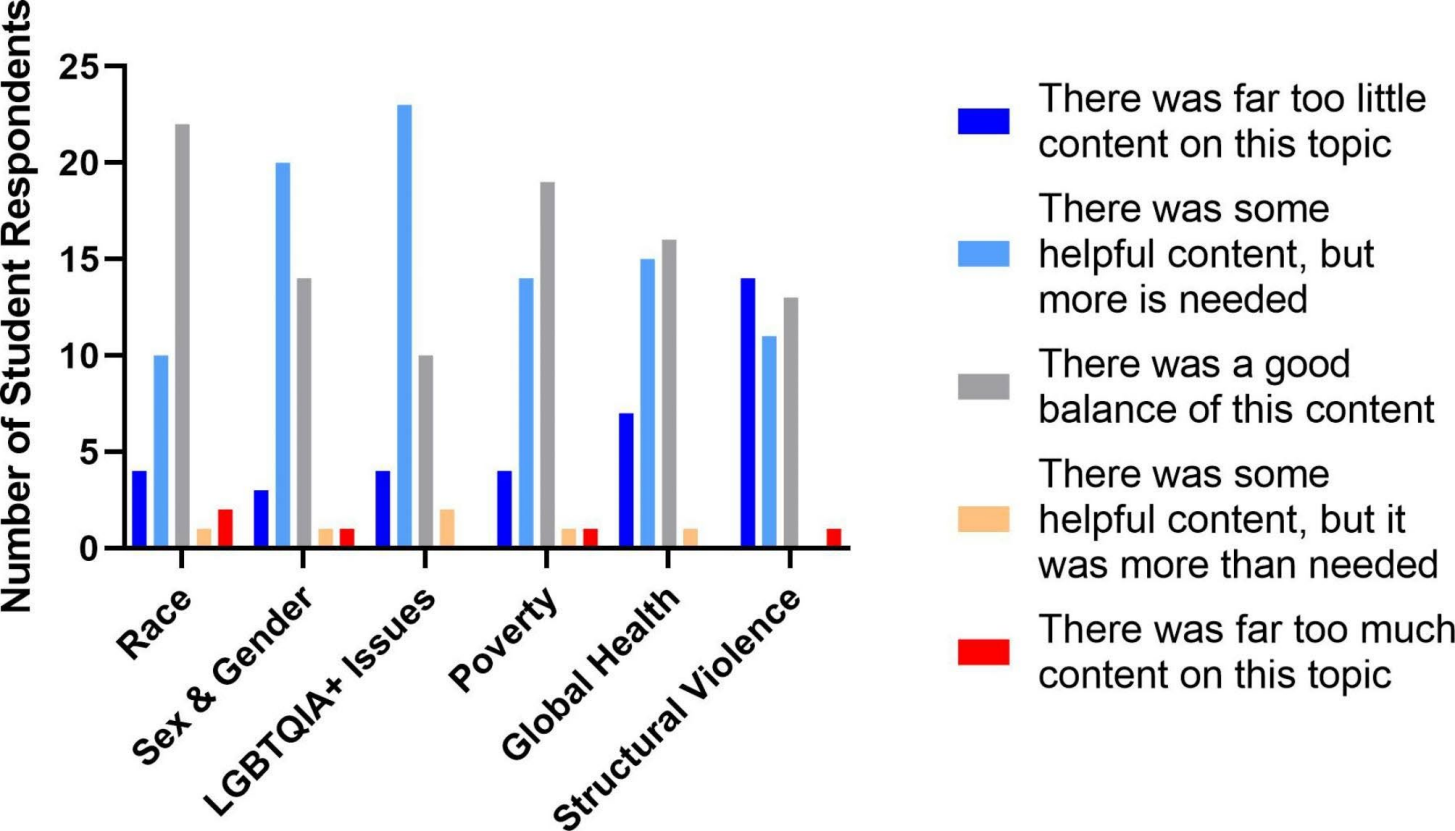
Student Opinions on Balance of SDH Topics in Curriculum. Students responded to Likert scale questions that gauged their perception on whether topics that contribute to SDH inequities are covered appropriately in the preclinical curriculum. (n = 40)

Students responded positively to the combination of infographic format and in-class announcements (Fig. 1). The majority of students (82.5%) indicated preference for the infographic format over other traditional formats, such as plain text or PowerPoint. Of the elements included in the infographics, articles played a role for most students (72.5%) in learning about SDH. Most students expressed that they were more interested in the related SDH due to announcements (80%) and that their knowledge of SDH had improved due to the SMTW (77.5%). Most students reported a “good balance” of race (65%), sex and gender (52.5%) and poverty (62.5%) in the infographics, while students reported too little content on LGBTQ + issues (57.5%), global health (52.5%), and structural violence (55%) (Fig. 2).

**Table 1:** Student Attitudes towards Social Medicine Curriculum and SMTW

Survey Question	Responses
	N (%)
Are you aware of the Social Medicine Theme of the Week?	
Yes	40 (100%)
How helpful did you find the Social Medicine Theme of the Week in synthesizing information regarding Social Determinants of Health into coursework and PCR?	
Very helpful	23 (57.5%)
A little helpful	14 (35.0%)
Not helpful at all	3 (7.5%)
How would you rate the extent to which you agree or disagree with the following statements?	
My knowledge of Social Determinants of Health and Social Medicine has increased in my first year at the Larner College of Medicine.	
Strongly agree	8 (20.0%)
Somewhat agree	21 (52.5%)
Neither agree nor disagree	8 (20.0%)
Slightly disagree	1 (2.5%)
Strongly disagree	2 (5.0%)
There should be more sessions or learning opportunities to address Social Determinants of Health	
Strongly agree	21 (52.5%)
Somewhat agree	11 (27.5%)
Neither agree nor disagree	7 (17.5%)
Slightly disagree	0 (0.0%)
Strongly disagree	1 (2.5%)
Did you interact with any Social Medicine Theme of the Week infographic?	
Yes	33 (82.5%)

#### Faculty

The response rate among faculty was low, with 33 (12.6%) respondents from 261 total faculty surveyed **(**Table 2). Most faculty respondents (64%) had heard of the SMTW and were aware of what is involved. Four (12%) had no knowledge of it. A majority (73%) reported that the SMTW was helpful in synthesizing information regarding SDH into coursework. Methods by which they incorporated related SDH into session content was by creating new slides or pre-reading (14%), building on existing slides (23%), or mentioning content in class (26%). For most faculty (73%), this additional preparation took less than 30 min.

**Table 2:** Faculty Attitudes towards Social Medicine Curriculum and SMTW

Survey Questions	Responses
	N (%)
Are you aware of the Social Medicine Theme of the Week?	
Yes	29 (87.9%)
How helpful did you find the Social Medicine Theme of the Week in synthesizing information regarding Social Determinants of Health into coursework?	
Very helpful	9 (27.3%)
A little helpful	15 (45.5%)
Not helpful at all	9 (27.3%)
How did you incorporate teaching about Social Determinants of Health content into your session(s)? (Check all that apply)	
New slides or pre-reading	6 (14.0%)
Built on existing slides or pre-reading	10 (23.3%)
Mentioned content in class but did not create new material	11 (25.6%)
Reviewed case presentations	5 (11.6%)
Created discussion questions	4 (9.3%)
How challenging did you find it to incorporate teaching about Social Determinants of Health into your course?	
Very challenging	3 (9.1%)
A little challenging	17 (51.5%)
Not challenging at all	13 (39.4%)
Were there any challenges that you faced in developing or delivering this content? (Check all that apply)	
I did not have prior experience with this topic	5 (11.4%)
I did not feel like I had adequate training in developing or delivering this content	11 (25.0%)
I did not know how to make the material fit into the academic learning objectives	4 (9.1%)
I was worried about saying something offensive	5 (11.4%)
I did not know how to mediate conflict surrounding the topic	4 (9.1%)
Did you feel you were able to successfully incorporate the Social Medicine Theme of the Week into your teaching?	
Yes	17 (51.5%)
Did you interact with any Social Medicine Theme of the Week infographic?	
Yes	14 (42.4%)

**Fig. 3:**
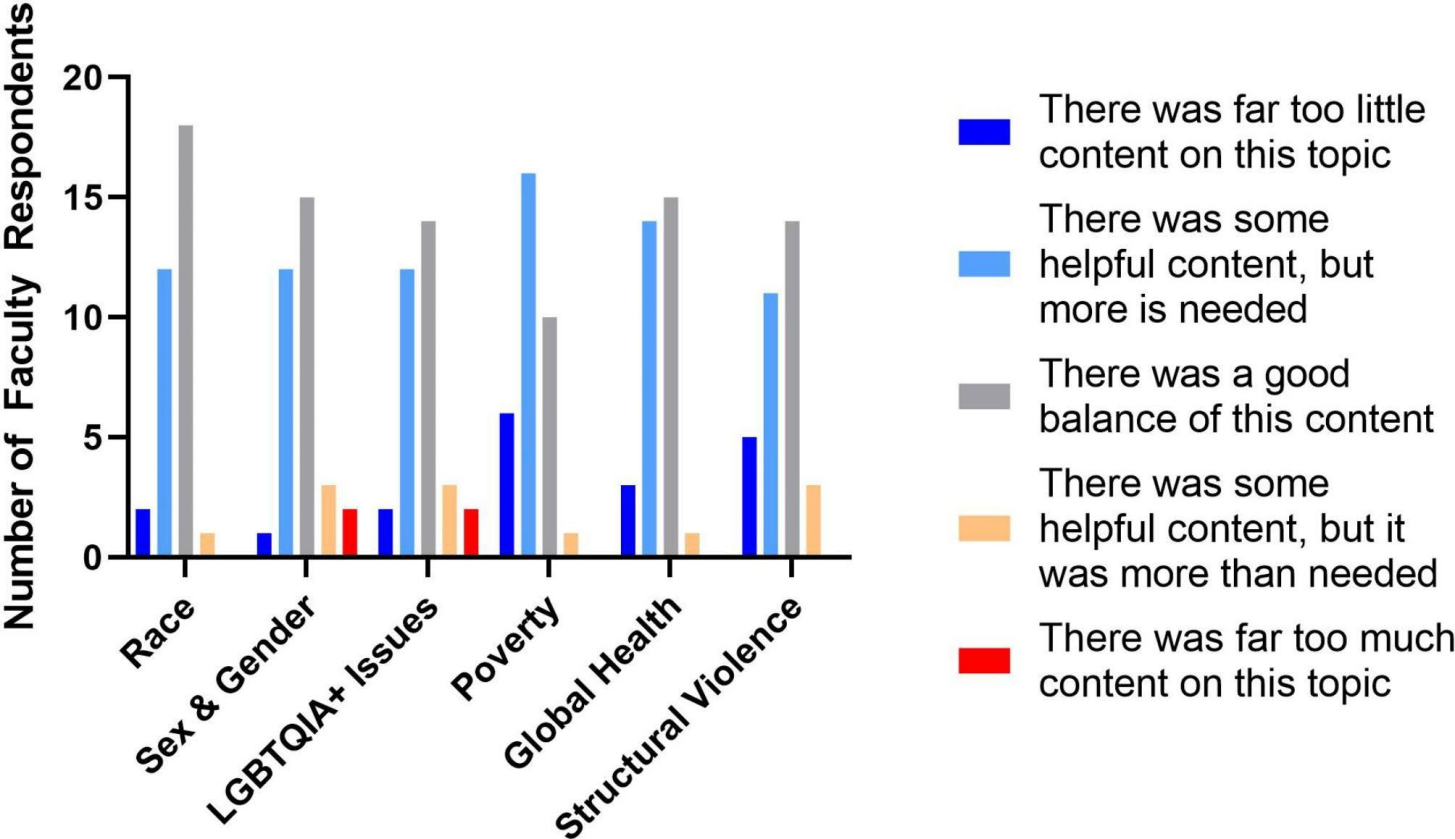
Faculty Opinions on Balance of SDH Topics in Curriculum. Faculty members responded to Likert scale questions that gauged their perception on whether topics that contribute to SDH inequities are covered appropriately in the preclinical curriculum. (n = 33)

Most faculty respondents indicated a “good balance” of all social medicine topics surveyed: race, sex & gender, LGBTQ + issues, poverty, global health, and structural violence (Fig. 3).

Topics where faculty reported too little content included poverty (67%), global health (52%), and structural violence (48%). Most respondents (61%) found it challenging to incorporate teaching about SDH into their course, and the most common reason behind this difficulty was feeling like they did not have adequate training to deliver the content (25%).

Faculty engagement with the SMTW and the related infographic was modest. The majority (58%) had not interacted with the infographic, and many (48%) reported that they felt they were not able to successfully incorporate the SMTW into their teaching. Engaging faculty in the SMTW is an area that needs improvement at our college and represents an important aspect of creating a successful integrated preclinical social medicine curriculum.

Qualitative Survey Results.

#### Student comments

Qualitative analyses indicate student desire for more active integration of SDH topics into coursework. Students cited specific examples of successful implementation of SDH material into coursework.

I thought that discussing obesity … was approached thoughtfully in the curriculum, and the [session] on addressing weight with patients allowed for discussion about body image and obesity.

Students indicated several specific Social Medicine or SDH topics that they learned about as a direct result of the SMTW:

The Social Medicine Theme of the Week reinforced my understanding of how intersection identities such as race, gender, sexual orientation, and class can collectively impact an individual’s access to societal resources.

The racial disparities in colorectal cancer Social Medicine Theme of the Week stands out to me the most, as this is something I was previously unaware of. I thought the infographic was effective and appreciated how it aligned with what we were learning in class.

Students provided salient suggestions for improvement of the Social Medicine Curriculum:

I would like to have the SMTW incorporated in the [curriculum], and I would like to have an open forum [to discuss issues] as a group.

#### Faculty comments

Faculty respondents voiced support for a more active integration of social medicine topics into the curriculum:

[Social determinants of health] should be considered in discussion of disease prevalence, diagnosis, therapeutics, and treatment as well as prevention.

[Social determinants of health] create important [connections] between social issues and all aspects of healthcare, from delivery, access, affordability, etc.

Would be nice to see integration into curriculum, including tested material that integrates clinical teaching and impact of social determinants.

When asked about an aspect of the SMTW that facilitated teaching, faculty shed light on its student-led nature:

The fact that it’s led by students, pitching the relevancy of content to peers, is powerful and well-aligned to the core message of shaking the foundations.

## Discussion

### Innovation and strengths

The novelty of the approach presented here is the use of student-designed infographics to integrate social medicine topics into the preclinical medical curriculum. This method gives rise to a middle-out approach to pedagogy and learning that incorporates and truly engages with students rather than top-down initiatives that may be removed from real-world contexts or concurrent class information. Where previous efforts have been limited to single curricular sessions, such as module-based learning and social medicine sessions created for specific clerkships, the SMTW focuses on longitudinal themes that apply to the topics covered in preclinical coursework [17, 18]. Some other projects may also focus on longitudinal multi-disciplinary curricular structures to educate students about social determinants of health, which the SMTW builds on by introducing the self- and peer-teaching component provided by infographic generation [19]. The evaluation of this approach suggests that the SMTW student-led pedagogical modality using student announcements and infographics is an effective way to engage students in SDH principles by directly linking them to the preclinical medical curriculum.

SMTW infographics were a well-received pedagogical tool. Students preferred the infographic approach over other pedagogical modalities and viewed the impact of SMTW infographics on SDH training favorably. Students reported an interest in the expansion of learning opportunities addressing SDH (Table 1), clearly indicating a growing collective consciousness regarding social issues and their impact on the practice of medicine. All student respondents were aware of the SMTW, and the majority found the complementary curriculum helpful for synthesizing SDH information. Additionally, a large proportion of student respondents reported their knowledge of SDH had increased during their first year of medical school.

The enthusiastic student engagement with alternative teaching modalities aligns with previous studies regarding student perceptions of infographics. Post-secondary education students overwhelmingly prefer infographics to plain text and perceive them to be a more engaging modality that facilitates learning at more rapid pace [20]. The use of student-created infographics in the medical setting can offer several unique benefits. Infographics deliver information more efficiently than narrative alone and have been shown to reduce cognitive load for students [15, 16]. The process of medical students creating infographics has also been demonstrated as an effective educational modality in itself, strengthening the information literacy, communicating science and data visualization skills of those who author them [15]. Infographics as a teaching modality for social medicine can allow students to engage social medicine topics no matter what level of background knowledge they possess.

The SMTW framework in the context of an integrated social medicine curriculum provides a variety of unique benefits for educators to consider [14]. In its current form, the SMTW relies heavily on student input for all aspects of the project; students are both the target audience and the creators of the educational material itself. Having students play an active role in developing their own curriculum can empower adult learners and engage students in curricular improvement processes that share traits with quality improvement methodologies used to create positive change in medicine [21]. Further, SMTW ameliorates the need for individual faculty buy-in or engagement with social medicine teaching, which is cited as the largest barrier preventing formal implementation of this material into medical curriculum [5]. Students are readily able to recognize the significance of SDH in relation to patient health and, as a result, are willing to generate the learning materials themselves.

### Limitations and opportunities

This work has limitations related to its ability to evaluate the impact of the SMTW curriculum on student learning and faculty teaching. The limited response rates, particularly for faculty surveys, may not represent the full spectrum of attitudes towards the SMTW and limit generalizability of the results. Intrinsic to survey studies as a research methodology is a limitation in verifiability and generalizability, which are compounded by the restricted sample size available (in this case, students and faculty who have been exposed to the SMTW). Students and faculty with high interest in social medicine education may have self-selected in responding to the survey, skewing responses toward the positive. Further, these responses represent respondents’ perceptions of the SMTW and their own knowledge, which may be affected by a variety of external factors such as time, recency bias, and incomplete knowledge, among others.

The SMTW is not yet formally assessed on exams, thus students may only passively engage with the material. Without formal assessment of the impact of SMTW on student skills or practice, there is no data available to evaluate whether SMTW components effectively teach and ingrain social medicine principles, nor can we evaluate whether the SMTW impacts achievement of relevant clinical competencies. This limitation is offset by the integration of the SMTW into curriculum that is assessed in the corresponding ethics and professionalism coursework, and there are opportunities to better define those integrated elements within assessments to include SMTW objectives.

Student involvement in the design and delivery of SMTW and social medicine infographics as well as other aspects of the social medicine curriculum has been a positive experience for both students and faculty. However, faculty engagement with the project beyond the core teaching faculty is limited, which impedes the ability of SMTW infographics to integrate social medicine teaching with other material in the preclinical curriculum. Inadequate faculty orientation to the SMTW as well as generational differences in perceptions of the role of physicians, and differing levels of investment in curriculum development may provide insight into the differences in enthusiasm [5]. A student-centered intervention that engages faculty participation too weakly can also lead to variable teaching materials that are insufficiently linked to the core curriculum and its assessment. The evaluation of the SMTW curriculum has identified opportunities to better engage faculty in the SMTW process, including earlier communication and providing faculty training in SDH topics before integrating them into sessions.

An important issue to consider is whether student-led approaches place the burden of educating peers or faculty members on students who are personally affected by SDH. Without adequate support, this voluntary system easily can morph from inclusive teaching into an egregious example of the “minority tax” levied on students and faculty of color in academic medicine [22]. To mitigate this risk, a core group of faculty champions, some with institutional support, oversee and deliver social medicine teaching. Student participants in the SMTW are compensated with visible authorship on the infographics and have access to mentored educational scholarship opportunities such as this paper and its predecessors [13, 14]. Regardless of these resources, curricular processes must be complemented by institutional investments in structural changes that promote diversity, equity and inclusion for students, faculty and staff members.

Several future opportunities were identified by evaluating this social medicine teaching initiative including (1) improvements in communication through earlier promotion of SMTW material to students and faculty and the creation of a centralized resource page for easy access; (2) improved assessment by formally embedding SMTW objectives into coursework to directly evaluate competency; (3) integration of this curriculum into the clinical curriculum and larger social medicine projects that support the institutional mission. With these forward-looking strategies, the SMTW will be afforded the visibility and measured outcomes it needs to succeed.

## Electronic supplementary material

Below is the link to the electronic supplementary material.

Appendix A

Appendix B

Appendix C

Appendix D

## Data Availability

The datasets used and/or analyzed during the current study are available from the corresponding author on reasonable request.
