# Supplementary material for: Integrating social determinants of health principles into the preclinical medical curriculum via student-led pedagogical modalities
Source: BMC Med Educ. 2023 Apr 4;23:210. doi: 10.1186/s12909-023-04152-0 (PMC10072025; doi:10.1186/s12909-023-04152-0)
Supplement: Supplementary file 1 — Appendix A [file 12909_2023_4152_MOESM1_ESM.docx]

The pre-clinical curriculum has ethics topics embedded into the basic science courses and two longitudinal courses that focus on Professionalism and Public Health Projects respectively. The SMTW is used to emphasize the connections between the basic science topics and the ethics, professionalism, and public health topics.

# **Appendix A: Alignment of weekly SMTW topics with Pre-clinical Basic Science, Ethics, Professionalism and Public Health**

| Course | SMTW Topic (hyperlink to Infographic) | Basic Science/Ethics Topic | Professionalism/Public Health |
| --- | --- | --- | --- |
|  | *Fall MS1* |  |  |
| Foundations of Clinical Sciences | Introduction to SMTW | Doctoring Skills; Cell Signaling; Intro to Ethics | Personal role in Medicine |
|  | Advocacy for Vulnerable Populations | Evidence-based medicine; Diagnostic/Genetic testing; Biochemistry | Tenets of Professionalism |
|  | Death and Dying | Gross Anatomy; Cell Signaling; Ethics Medical Decision Making | Cadaver and Mortality |
|  | Physician Advocacy | Muscle Physiology; Biochemistry; Ethics of Autonomy | Giving and Receiving Feedback |
|  | Providing Hope and Humility | Genetics; Cell Cycle and DNA repair; Pharmacogenomics | Being Present |
|  | Power and Privilege | Cell Death; Cancer; LGBTQ Health Equity; White Coat Ceremony | Power and Privilege |
|  | Trauma-informed Care | Interprofessional Health Care Teams; Immune system | Adverse Childhood Experiences |
|  | Cultural influences on Health Care Decisions | Cardiovascular System; End of Life Ethics | Patient Stories 1 |
|  | Medicine, Race, and Implicit Bias | Renal System; Pediatric Ethics | Implicit Bias |
|  | Narrative Medicine | Gastrointestinal tract; Ethics of Race and Culture | Language and Medicine |
|  | Social Determinants of Abortion | Reproductive System; Gender-affirming patient communication; Genetic testing; Inclusive Sexual Ethics | Empathy |
|  | Stress and Gender Identity | Endocrinology; Ethics Wellness and Humility | Patient Stories 2 |
|  | *Spring MS1* |  |  |
| Attacks & Defenses | [Racial Disparities in Blood Disorders](https://venngage.net/ps/ZLYgMDorA4M/smtw-racial-disparities-in-blood-disorders) | Sickle Cell Anemia; Bleeding Disorders | Stigma and Discrimination 1 |
|  | [History and Impact of Vaccines](https://venngage.net/ps/vJAM8cqVgiU/smtw-history-and-impact-of-vaccines-in-america) | Immunology | Stigma and Discrimination 2 |
|  | [History of Racism and Discrimination in Medicine](https://venngage.net/ps/WVsZjwecxs/smtw-history-of-racism-in-medicine) | Bacteriology; Antibiotics | Stigma and Discrimination 3 |
|  | [The AIDS Crisis](https://venngage.net/pl/dVBMuQ95YGs) | Virology | Social Determinants of Health |
|  | [Racial Disparities in COVID-19](https://venngage.net/ps/efV4igyJ82o/smtow-covid-19-disparities) | Inflammation and Cancer | Communities 1; Intro to Public Health |
| Nutrition, Metabolism, & Gastrointestinal System | [Social Determinants and Disparities in Nutrition](https://venngage.net/ps/jTTEAS9XMSo/smtw-health-disparities-in-nutrition) | Digestion; vitamins; metabolism | Communities 2 |
|  | [Racial Disparities in Colorectal Cancer](https://venngage.net/ps/RgYjei8jNgw/smtw-racial-disparities-in-colorectal-cancer) | Microbiome; Colorectal Cancer | Public Health Projects |
|  | [Access to Food and Water](https://venngage.net/ps/M0hH48aXyM0/smtw-access-to-food-and-water) | Micronutrients; Infectious Diarrhea | Public Health Projects |
|  | [Physician Burnout](https://venngage.net/ps/MYthB6OCHFE/smtw-physician-burnout) | Abdominal Physical Exam; metabolism | Public Health Projects |
|  | [Food as Medicine](https://venngage.net/ps/RGKyIdJKe0U/smtw-food-as-medicine) | Cholesterol Disorders | Culture and Spirituality |
|  | [Social Determinants of Diabetes](https://venngage.net/ps/rJg5XFzDWlk/smtw-social-determinants-of-diabetes) | Type I and Type II Diabetes | Public Health Projects |
|  | [Body Image and Obesity](https://venngage.net/pl/Pc2uTdpIaw0) | Thyroid Disorders | Public Health Projects |
| Medical Neuroscience | [Rural Health](https://venngage.net/ps/4Au6TRxF8k/lcomsmtw_ruralhealth_2021) | Autonomic Nervous System; | Public Health Projects |
|  | [Mental Health Disparities](https://venngage.net/ps/TXUx3T6FU0/smtw-mental-health-disparities) | Spinal Cord; CNS infections | Health Systems |
|  | [Allocation of Resources in American Healthcare](https://venngage.net/ps/aTmaoRYI3Mw/health-care-allocation-infographic) | Demyelinating Diseases; Ophthalmology | Public Health Projects |
|  | [Defining Disability](https://infograph.venngage.com/ps/UTnoVr49WVo/defining-disability-smtw) | Pain; Hearing Disorders | Public Health Projects; Disability Panel |
|  | [Aging and Bias](https://venngage.net/ps/R9FnHujZvA8/aging-and-medicine) | Movement Disorders; Stroke | Public Health Projects |
|  | [Neurodivergence and Neurodiversity](https://venngage.net/ps/AODeIXleApM/smtw-neurodiversity) | Cognition; Neurodegeneration | Public Health Projects Presentations |
|  | [Stigma and Mental Health](https://venngage.net/pl/0r2BUq1DEcY) | Psychiatry | Values and Health |
|  | Fall MS2 |  |  |
| Connections | [Social Determinants of Skin](https://venngage.net/ps/hBvcadAmgHI/social-determinants-of-skin-conditions) | Dermatology | Intro to Advocacy |
|  | [Social Determinants of Rheumatic Diseases](https://venngage.net/ps/yzrZwvmaFT8/smtw-social-determinants-of-rheumatic-diseases) | Rheumatology |  |
|  | [Child Abuse](https://venngage.net/ps/BBOLEohNVg/smtw-child-abuse) | Orthopedics; Pediatric Injuries | Advocacy for Patients |
| Cardiovascular, Respiratory, & Renal Systems | [Atherosclerosis Prevention](https://venngage.net/ps/WgwaKMfmRjc/smtw-atherosclerosis-prevention) | ECGs; Valvular Diseases | Optional 5k Run/hike |
|  | [Social Determinants of Cardiovascular Disease](https://venngage.net/pl/Tmoz6k5r2P0) | Hypertension; Heart Disease | Careers in Medicine |
|  | [Chagas Disease and Immigrant Health](https://venngage.net/ps/wynOnGqbLY/smtw-chagas-disease-and-immigrant-health) | Cardiomyopathies | Global Health Opportunities |
|  | [Social Determinants of Respiratory Disease](https://infograph.venngage.com/pl/fPlLbRImIg) | Pulmonary Pathophysiology | Advocacy for Colleagues |
|  | [Perspective on Race-based eGFR](https://venngage.net/ps/sfilTqqMTCQ/smtw-brief-perspective-on-race-based-egfr) | Renal Disorders | Institutional-level Advocacy |
| Human Development & Reproductive Health | [Sexual Health and Stigma](https://venngage.net/ps/UVhN4CYcSy0/smtw-sexual-health-stigma) | Reproductive organ pathophysiology | Transgender Patient Panel |
|  | [Bodily and Sexual Autonomy](https://venngage.net/ps/BMEBtTPIWhs/bodily-and-sexual-autonomy) | Pregnancy | Population-level Advocacy |
|  | [Social Determinants of Learning](https://venngage.net/ps/wQyiaOayRIg/smtw-social-determinants-of-learning) | Childhood and Adolescence; Intellectual disability | Allyship and Advocacy |
|  | [Aging Well](https://infograph.venngage.com/pl/VqjAIiLpAfs) | Geriatrics | Specialized Care for Older People |
