# Supplementary material for: Integrating social determinants of health principles into the preclinical medical curriculum via student-led pedagogical modalities
Source: BMC Med Educ. 2023 Apr 4;23:210. doi: 10.1186/s12909-023-04152-0 (PMC10072025; doi:10.1186/s12909-023-04152-0)
Supplement: Supplementary file 2 — Appendix B [file 12909_2023_4152_MOESM2_ESM.pdf]

SMTW: Racial Disparities & Covid

Presented by UVM Larner Social Justice Coalition

CDC COVID-NET March 2020 Data

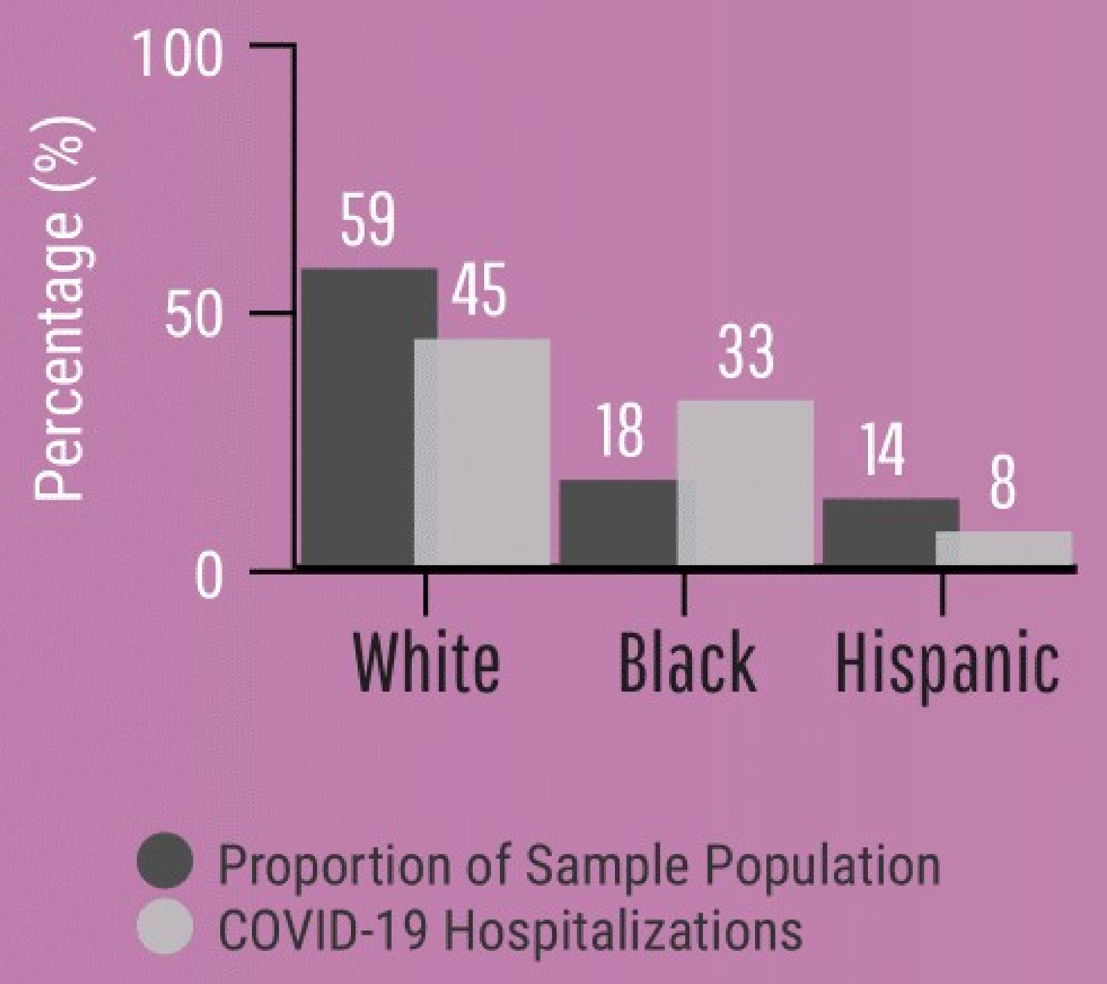

Garg S, Kim L, Whitaker M, et al. Hospitalization Rates and Characteristics of Patients Hospitalized with Laboratory-Confirmed Coronavirus Disease 2019 — COVID-NET, 14 States, March 1–30, 2020. MMWR Morb Mortal Wkly Rep 2020;69:458–464. DOI: http://dx.doi.org/10.15585/mmwr.mm6915e3

The goal of this weeks newsletter is to explore the effects of Covid-19 on the health of racial and ethnic minority groups as well as the underlying health disparities contributing to the disproportionate burden of illness and death among these populations. Though data is limited, early estimates from the CDC (depicted graphically to the right), suggest an **overrepresentation of African-Americans among hospitalized patients**. Additionally, preliminary NYC data identified death rates among Black/African American persons (92.3 deaths/100,000) & Hispanic/Latino persons (74.3) to be substantially higher than those of White (45.2) or Asian (34.5) persons.

So why does race/ethnicity seem to have a significant influence on Covid-19 outcomes?
As you can imagine, the answer is complicated. The increased risk of complications and adverse outcomes were seeing among African-American and Hispanic/Latino patients is likely the due to an increased susceptibility to disease as a result of pre-existing chronic health conditions. The March 2020 CDC data approximated that 90% of hospitalized Covid-19 patients had one or more underlying conditions, the most common being **obesity, hypertension, chronic lung disease, diabetes mellitus, and cardiovascular disease.** Minority populations experience significant disparities in regards to these chronic conditions, the underlying causes of which have been linked to **genetics, lack of economic resources, limited access to health care, delay in treatment, cultural beliefs, as well as environmental factors including micro-aggressions, racism, and discrimination.** Over time, these stressors increase the incidence of chronic disease by eliciting a heightened baseline sympathetic response in the individuals experiencing them, leading to elevated levels of stress hormones in the bloodstream.

The racial disparities indicated in Covid-19 are the result of the dynamic interplay of not only pre-existing conditions, but also the social determinants of health that contribute to those underlying conditions. We encourage you to explore the multi-media resources below related to these topics to further develop your understanding of these disparities as well as their underlying risk factors.

Who's Hit Hardest By COVID-19? Why Obesity, Stress And Race All Matter

How racism and micro-aggressions lead to worse health outcomes

Reported Racial Discrimination, Trust in Physicians, and Medication Adherence Among Inner-City African Americans With Hypertension

AHA: Coronavirus precautions for patients and others facing higher risks

Statistics obtained from:
CDC: Coronavirus Disease 2019, Racial and Ethnic Minority Groups

Please send any feedback to
krisandra.kneer@med.uvm.edu

ABC NEWS: Coronavirus is disproportionately killing the black community. Here's what experts say can be done about it

ABC NEWS story interviewing COVID-19 patients & physicians in areas of country where disparities are most apparent and severe

Ted Talk: How racism makes us sick

David R. Williams explains the impact of discrimination on well-being, going beyond traditional measures to reveal how factors like implicit bias, residential segregation and negative stereotypes create and sustain inequality.

PBS NEWS: COVID-19 may not discriminate based on race — but U.S. health care does

Amna Nawaz talks to Dr. Uché Blackstock of Advancing Health Equity about the disparities long present in the US medical system that are driving a crisis within a crisis among black and brown communities.

NPR: This Racism is Killing Me Inside

Discusses how micro-aggressions and racial discrimination evoke an underlying physiological sympathetic response that leads to chronic stress and increased susceptibility to adverse health outcomes.

NPR: The News Beyond The COVID Numbers

Code Switch episode that discusses the importance of tracking the coronavirus's impact on different racial groups, and how to translate that data into actual improvements in health disparities.

CNN: Dr. Sunjay Gupta;s Coronavirus: Fact vs. Fiction podcast

CNN's Chief Medical correspondent Dr. Sanjay Gupta breaks down the various factors and demographics that may make some people and communities more susceptible.

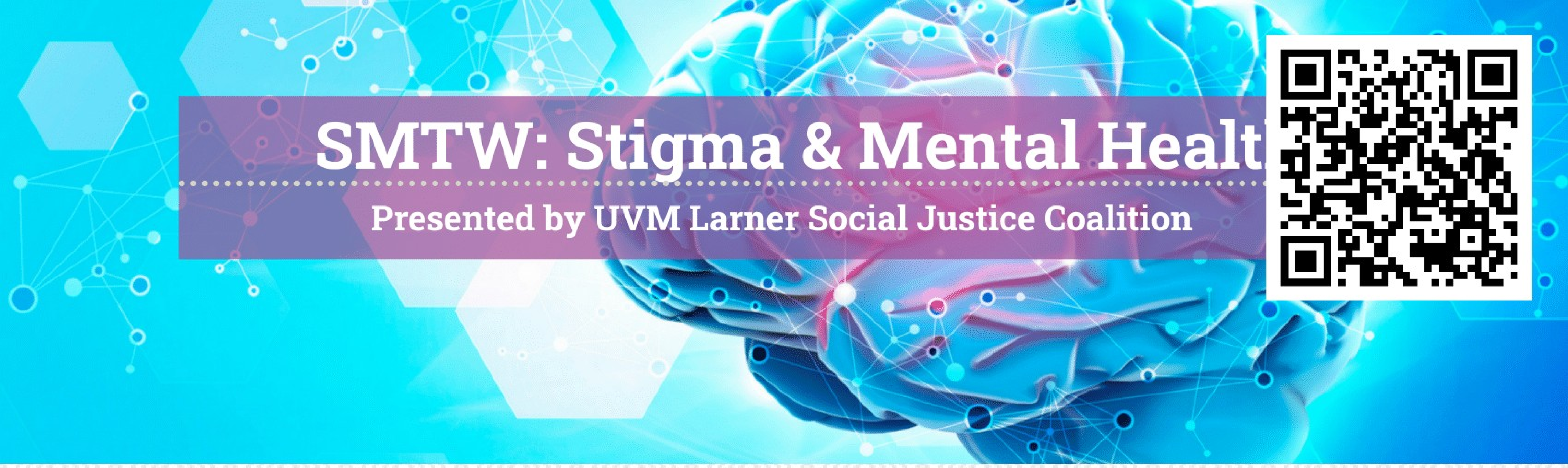

SMTW: Stigma & Mental Health

Presented by UVM Larner Social Justice Coalition

Many people with mental illness, especially serious mental illness, are challenged two-fold. On one hand, they struggle with the symptoms of their diagnosis, on the other they are challenged by the societal misconceptions about mental illness. Both public and self-stigma may be understood in terms of three components: **stereotypes, prejudice, & discrimination**. As a result, individuals with mental illness face disproportionate barriers to the opportunities that define a quality life: good jobs, safe housing, as well as satisfactory health care. We encourage you this week to explore the resources provided to expand your understanding of stigma and consider the ways in which you as a future provider can combat these misconceptions about mental health.

Themes of misconceptions about mental illness & their corresponding stigmatizing attitudes

**Fear & Exclusion**	Persons with severe mental illness should be feared and, therefore, be kept out of most communities
**Authoritarianism**	Persons with severe mental illness are irresponsible, so life decisions should be made by others
**Benevolence**	Persons with severe mental illness are childlike and need to be cared for

(World Psychiatry 2002)

Prevalence

- 1 in 5 U.S. adults experience mental illness
- **1 in 25** — 1 in 25 U.S. adults experience serious mental illness
- **>50%** of U.S. adults will experience a mental illness in their lifetime

Treatment

- **43%** of U.S. adults with mental illness who receive treatment in a given year
- **11 YEARS** Average delay between symptom onset & treatment
- **64%** of U.S. adults with serious mental illness who receive treatment in a given year

(NIMH 2018)

9 Ways to Fight Mental Health Stigma

Could COVID-19 Finally De-stigmatize Mental Illness?

The Catastrophic Effects of Mental Health Stigma

Untreated Minds: 3 Barriers to Mental Health Services That Impact Millions

Halsey: A Conversation About Bipolar Disorder

Halsey and licensed therapist Dr. Snehi Kapur explore mental health and bipolar disorder in an intimate one-on-one conversation

Ted Talk: A Tale of Mental Illness

In this powerful talk, Elyn Saks asks us to see people with mental illness clearly, honestly and compassionately.

Stigma Podcast: Mental Health

Podcast series by Stephan Hays who is a recovering addict, living with type-1 bipolar disorder. "On this show you will hear stories of addiction, mental illness, recovery, hope, and wellness."

Stigmatized Podcast

"Our goal at Stigmatized is to build a community that will deliver awareness, hope, and healing for those that have been cast aside for far too long."

Please send any infographic feedback to krisandra.kneer@med.uvm.edu

SMTW: The AIDS Crisis

Presented by UVM Larner Social Justice Coalition

HIV (human immunodeficiency virus) is an STI that destroys CD4+ T cells. If left untreated, it can lead to AIDS (acquired immunodeficiency syndrome), which, without treatment, can be fatal in about three years. HIV can be spread through contact with certain bodily fluids of a person with HIV, unprotected sex, or sharing drug injection supplies. In 1981, the disease later known as AIDS was reported on as a "rare cancer" affecting homosexuals. As AIDS continued to kill more and more people, the U.S. government ignored the epidemic and neglected to fund research on potential treatments, dismissing AIDS as a homosexual disease.

The AIDS Coalition to Unleash Power (ACT UP) was formed by activists to push for vital medical research into the disease. Their organizing work led to major changes in research and patient advocacy. The disproportionately high rates of HIV/AIDS among men who have sex with men (MSM) and black people continues today despite the availability of new therapies. Efforts to educate people about these conditions and combat stigma around HIV/AIDS have not fully addressed the myriad barriers to HIV prevention, especially among MSM and people of color.

Health Disparities

- Today, around **1.2 million people** are living with HIV in the United States. While black MSM are the most affected by this condition, other factors can also put people at greater risk of HIV.
- Transgender people experience high levels of stigma and violence and the inadequate access to affirming health care leads to high HIV rates.
- Free HIV testing, antiretroviral therapies, and pre-exposure prophylaxis (PrEP) are becoming more accessible, but more effort is needed to address HIV stigma, distrust of the medical system, and additional barriers to receiving good quality health care.

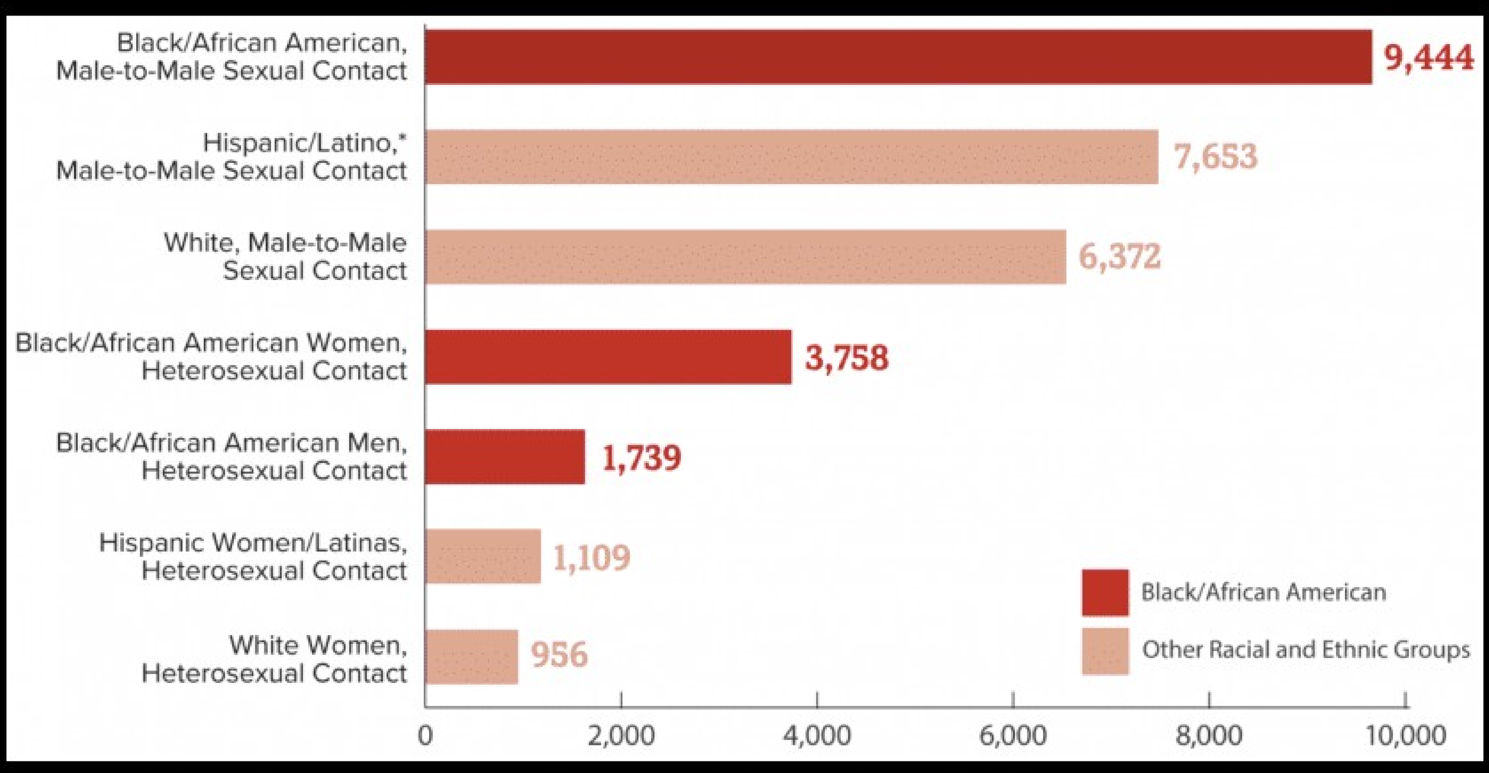

Diagnoses of HIV infection in the United States and dependent areas, 2018

1981
AIDS was officially recognized as a distinct disease. Because the men getting this rare lung infection were homosexual, doctors assumed the cause was the "homosexual lifestyle." By the end of that year, there were 270 confirmed cases and 120 deaths.

1987
ACT UP held its first action on Wall Street to protest the profiteering of pharmaceutical companies on AIDS drugs (especially Burroughs Wellcome, manufacturer of AZT).

1990
ACT UP protests at the National Institutes of Health. The group demands more HIV treatments and the expansion of clinical trials to include more women and people of color.

1997
New HIV infections peaked at an estimated **3.5 million** new cases despite at 47% drop in HIV/AIDS related deaths. HIV transmission remained a major problem as antiretroviral therapy access improved.

2003
ACT UP and Health Gap members returned a "bounced check" for $15 billion to the Bush re-election campaign, representing the money they promised to fight global AIDS but failed to deliver.

2015
CDC's annual HIV Surveillance Report indicates that HIV diagnosis rates in the U.S. remained stable between 2009-2013, but MSM, young adults, racial/ethnic minorities, and individuals living in the South continue to bear a disproportionate burden of HIV.

2018
The U.S. Preventive Services Task Force issues a draft recommendation that clinicians should offer PrEP to individuals at high risk for HIV infection.

Shame and HIV: Strategies for addressing the negative impact shame has on public health and diagnosis and treatment of HIV

In this Pandemic, Personal Echoes of the AIDS Crisis

HIV AND AIDS IN THE UNITED STATES OF AMERICA (USA)

The Silent HIV Crisis Sweeping the American South

How '80s AIDS Activist Group ACT UP Changed The Face Of Medicine : Shots - Health News : NPR

HIV Stigma: How Far We've Come, How Little We've Gained | Shawn Krueger | TEDxTallahassee

Queer Health Pod: The PrEPisode
Primary care physicians discussing the history of pre-exposure prophylaxis (PrEP) for HIV and who might benefit from PrEP

How Activists Were Finally Heard About The AIDS Epidemic : 1A : NPR

HIV/AIDS Statistics obtained from: Avert.org

Timeline information adapted from: Actupny.com, HIV.org, and Red.org

Infographic Contributors: Mahima Poreddy, Krisandra Kneer, Erik Zhang, Tyler Harkness

Please send any feedback to mahima.poreddy@med.uvm.edu

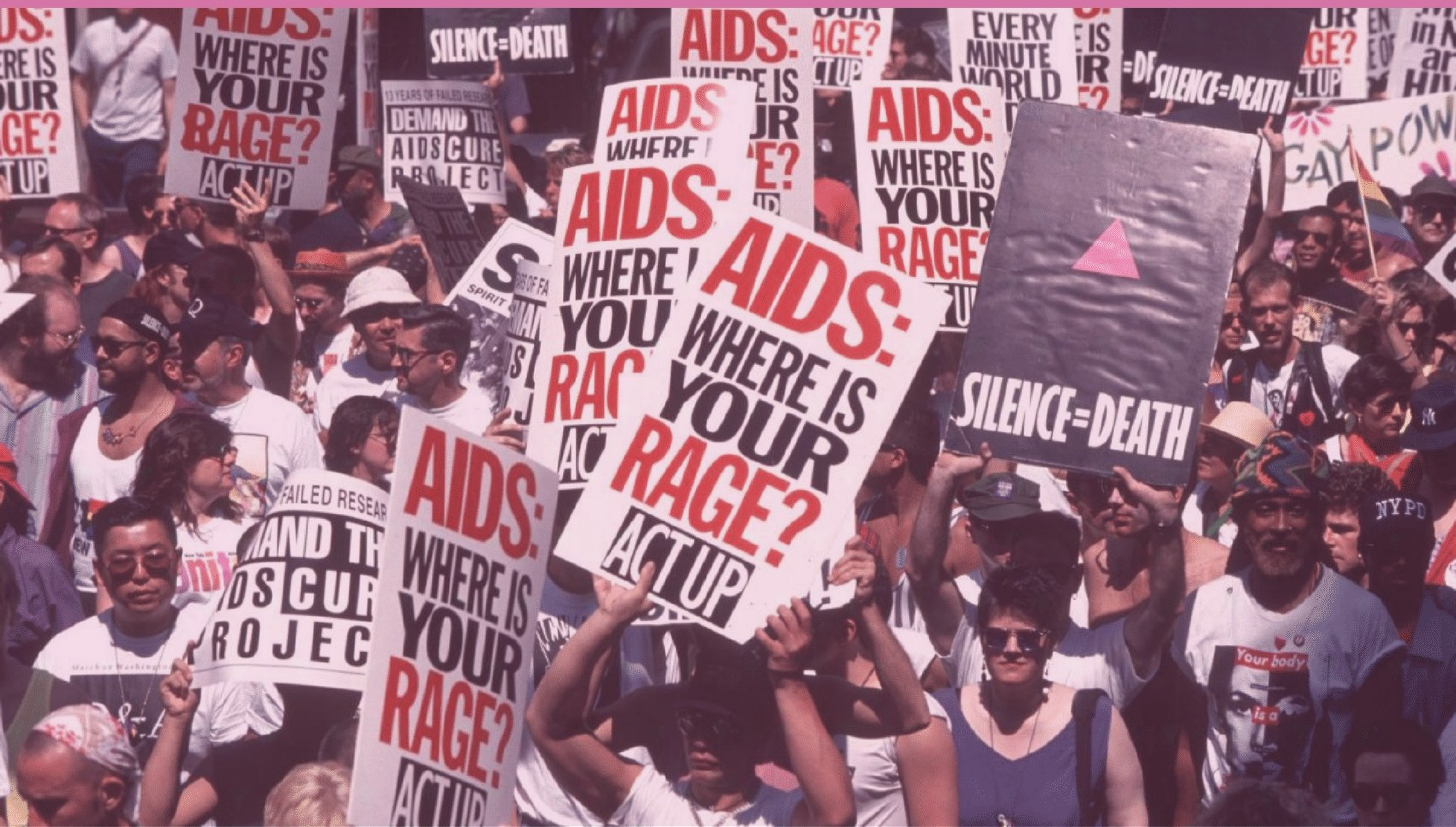

SMTW: Racial Disparities in Blood Disorders

Presented by UVM Social Justice Coalition

Associations between race and disease have long historical roots in America. Since colonial times, the prevalence and effect of syphilis, cholera, and pellagra, to name a few, have unjustly defined the health status of black communities - largely a result of the living conditions they were subjected to.

Blood disorders like G6PD deficiency, alpha thalassemia, and beta thalassemia also face the same racialized associations. Sickle cell disease (SCD) remains one of the most well-recognized of these disorders. Although treatment has improved over the past decades, people living with SCD (disproportionately black) face worse health outcomes and continue to receive limited institutional support.

100,000

Americans live with SCD

1 in 365

Black children are born with SCD.

The incidence for sickle cell trait is about 73.1 per 1000 black newborns; 3.0 per 1000 white newborns.

American patients with SCD have

34 years

less years of quality-adjusted life expectancy than the general population.

The estimated maximum annual cost for treatment of SCD is

$231,000

The estimated maximum annual cost for treatment of hemophilia was $35,000.

60%

of patients with SCD used Medicare or Medicaid as their primary payment option.

Most people living with SCD are publicly insured, and Medicaid recipients face substantial barriers in accessing specialized healthcare, which can drastically improve health outcomes in SCD cases.

Research funding from national foundations for cystic fibrosis (CF) was up to

440-fold

higher than funding for SCD research. Funding from the NIH was 3.5-fold higher per affected individual.

CF is another recessive genetic disease that primarily affects white people. Despite affecting far fewer Americans, CF research receives far more funding per patient than SCD research, which has likely contributed to numerous CF therapies receiving FDA approval in the same time that one drug was approved for SCD.

Sources: Centers for Disease Control and Prevention, "Data and Statistics on Sickle Cell Disease"; "Incidence of Sickle Cell Trait in the US"
Boston Medical Center, "Reducing Care Fragmentation in Sickle Cell Disease"

Infographic Contributors: Krisandra Kneer, Erik Zhang, Tyler Harkness
Please send any feedback to erik.zhang@med.uvm.edu

When Actions Speak Louder than Words -- Racism and Sickle Cell Disease

Health Disparities May Affect End-of-life Experiences of Minority Blood Cancer Patients

Why Are Therapeutics Still Lagging For Sickle Cell Disease? In Short, Systemic Racism

The Myth of Black Immunity: Racialized Disease During the COVID-19 Pandemic

Incomplete Cell
An amateur documentary directed by a sickle cell patient detailing the effect of sickle cell disease on patients and the communities they exist in.

Sickle Cell Disease: a battle for equality, justice and respect
Dr. Ahmar Zaidi details the struggles of one patient forced into homelessness by a healthcare system that unjustly treats its communities of color impacted by chronic disease.

We Had a Chance to Wipe Out Sickle Cell Disease. And, We Didn't
PBS's The Pulse addresses the failure to address sickle cell disease despite an increase in research funding in the 1970s.

Sickle Cell Disease: Invisible Illness, Enduring Strength
This Podcast Will Kill You covers the long-standing injustice and unending fight to raise awareness and provide support for those impacted by sickle cell disease.
