# Supplementary material for: Integrating social determinants of health principles into the preclinical medical curriculum via student-led pedagogical modalities
Source: BMC Med Educ. 2023 Apr 4;23:210. doi: 10.1186/s12909-023-04152-0 (PMC10072025; doi:10.1186/s12909-023-04152-0)
Supplement: Supplementary file 3 — Appendix C [file 12909_2023_4152_MOESM3_ESM.docx]

**Appendix C: Student Survey**

**Student Survey: Evaluation of the Social Medicine Theme of the Week**

Introduction: This survey aims to evaluate the Social Determinants of Health (SDoH) curriculum and Social Medicine Theme of the Week to identify key areas for curricular improvement and to understand the student and faculty experiences with SDoH material.

Your participation in this survey is optional, and you may stop participation at any time. We expect that completion of this survey should take less than 15 minutes. Your time is valuable; thank you for your help in improving and evaluating the SDoH curriculum.

All data collected will be analyzed and reported in aggregate. The survey is anonymous and we will not be able to match individual responses to email addresses or other identifiers. The results of this survey will contribute to both curriculum quality improvement and to our scholarly research projects.

By completing the survey, I affirm that I am a student at the Larner College of Medicine, and I agree to answer all questions to the best of my knowledge.

If you have any questions about this survey, feel free to contact Erik Zhang at Erik.Zhang@med.uvm.edu.

**SMTW Student Survey Questions**

Are you aware of the Social Medicine Theme of the Week?

- 1. Yes
  2. No

1. How helpful did you find the Social Medicine Theme of the Week in synthesizing information regarding Social Determinants of Health into coursework and PCR?
   1. Not helpful at all
   2. A little helpful
   3. Very helpful
2. How would you rate the balance of content about the following Social Determinants of Health in your first year of medical school at Larner?

|  | There was far too little content on this topic | There was some helpful content, but more is needed | There was a good balance of this content | There was some helpful content, but it was more than needed | There was far too much content on this topic |
| --- | --- | --- | --- | --- | --- |
| Race |  |  |  |  |  |
| Sex & gender |  |  |  |  |  |
| LGBTQ+ issues |  |  |  |  |  |
| Poverty |  |  |  |  |  |
| Global Health |  |  |  |  |  |
| Structural Violence |  |  |  |  |  |

1. How would you rate the extent to which you agree or disagree with the following statements?

|  | Strongly disagree | Slightly disagree | Neither agree nor disagree | Slightly agree | Strongly agree |
| --- | --- | --- | --- | --- | --- |
| My knowledge of Social Determinants of Health and Social Medicine have increased in my first year at Larner |  |  |  |  |  |
| There should be more sessions or learning opportunities to address Social Determinants of Health |  |  |  |  |  |

1. Please give a specific example of a time when Social Determinants of Health were taught well in the first year curriculum at Larner College of Medicine.
   1. Free text
2. Please give a specific example of a time when Social Determinants of Health could have been taught better in the first year curriculum at Larner College of Medicine.
   1. Free text
3. Did you help create an SMTW infographic?
   1. Yes
   2. No
4. Did you interact with any SMTW infographic?
   1. Yes
   2. No
5. How much did you engage with the following interactable elements of the infographic?

|  | I never engaged with this element | This element was unhelpful to my learning about Social Determinants of Health | This element played a minor role in my learning about Social Determinants of Health | This element played a major role in my learning about Social Determinants of Health |
| --- | --- | --- | --- | --- |
| Podcasts |  |  |  |  |
| Videos |  |  |  |  |
| Articles |  |  |  |  |

1. How would you rate the balance of content about the following Social Determinants of Health from the Social Medicine Theme of the Week?

|  | There was far too little content on this topic | There was some helpful content, but more is needed | There was a good balance of this content | There was some helpful content, but it was more than needed | There was far too much content on this topic |
| --- | --- | --- | --- | --- | --- |
| Race |  |  |  |  |  |
| Sex & gender |  |  |  |  |  |
| LGBTQ issues |  |  |  |  |  |
| Poverty |  |  |  |  |  |
| Global Health |  |  |  |  |  |
| Structural Violence |  |  |  |  |  |

1. I feel more knowledgeable about the following issues as a result of engaging with the SMTW infographics: (Check all that apply).
   1. Race
   2. Sex & gender
   3. LGBTQ issues
   4. Poverty
   5. Global Health
   6. Structural Violence
2. How would you rate the extent to which you agree or disagree with the following statements?

|  | Strongly disagree | Slightly disagree | Neither agree nor disagree | Slightly agree | Strongly agree |
| --- | --- | --- | --- | --- | --- |
| The infographic format was engaging |  |  |  |  |  |
| I prefer the infographic format over other traditional formats (plain text, PowerPoint) |  |  |  |  |  |
| The Social Medicine Theme of the Week announcements made me interested in learning more about the related Social Determinants of Health |  |  |  |  |  |
| My knowledge of Social Determinants of Health and Social Medicine have increased because of exposure to the Social Medicine Theme of the Week |  |  |  |  |  |
| Social Medicine Theme of the Week adequately addresses Social Determinants of Health |  |  |  |  |  |

1. Is there anything else you’d like to see included in the infographics?
   1. Free text
2. What was one principle or detail you learned about social medicine or the Social Determinants of Health from the Social Medicine Theme of the Week infographic?
   1. Free text
3. We are planning to use results from this survey to improve and modify social medicine teaching moving forward. Please use the space below to make suggestions or comments.
   1. Free text
