# Supplementary material for: Integrating social determinants of health principles into the preclinical medical curriculum via student-led pedagogical modalities
Source: BMC Med Educ. 2023 Apr 4;23:210. doi: 10.1186/s12909-023-04152-0 (PMC10072025; doi:10.1186/s12909-023-04152-0)
Supplement: Supplementary file 4 — Appendix D [file 12909_2023_4152_MOESM4_ESM.docx]

**Appendix D: Faculty Survey**

**Faculty Survey: Evaluation of the Social Medicine Theme of the Week**

Introduction: This survey aims to evaluate the Social Determinants of Health (SDoH) curriculum and Social Medicine Theme of the Week to identify key areas for curricular improvement and to understand the student and faculty experiences with SDoH material.

Your participation in this survey is optional, and you may stop participation at any time. We expect that completion of this survey should take less than 15 minutes. Your time is valuable; thank you for your help in improving and evaluating the SDoH curriculum.

All data collected will be analyzed and reported in aggregate. The survey is anonymous and we will not be able to match individual responses to email addresses or other identifiers. The results of this survey will contribute to both curriculum quality improvement and to our scholarly research projects.

By completing the survey, I affirm that I am a faculty member at the Larner College of Medicine, and I agree to answer all questions to the best of my knowledge.

If you have any questions about this survey, feel free to contact Erik Zhang at Erik.Zhang@med.uvm.edu.

**SMTW Faculty Survey Questions**

1. How would you describe your awareness of the Social Medicine Theme of the Week? (Please only choose one of the following)
   1. Never heard of it
   2. Heard of it but not sure what is involved
   3. Heard of it and aware of what is involved
2. How did you learn about the Social Medicine Theme of the Week? (Choose all that apply)
   1. Formal email from Dr. Lounsbury
   2. Formal email from Course Director
   3. Discovered on my own
   4. Heard from students
   5. Heard from another faculty member
   6. Other: _____
3. How would you rate the balance of content about the following Social Determinants of Health in the Foundations medical curriculum (years 1 and 2)?

|  | There was far too little content on this topic | There was some helpful content, but more is needed | There was a good balance of this content | There was some helpful content, but it was more than needed | There was far too much content on this topic |
| --- | --- | --- | --- | --- | --- |
| Race |  |  |  |  |  |
| Sex & gender |  |  |  |  |  |
| LGBTQ issues |  |  |  |  |  |
| Poverty |  |  |  |  |  |
| Global Health |  |  |  |  |  |
| Structural Violence |  |  |  |  |  |

1. Describe the role you believe the Social Determinants of Health should have, if at all, in the Foundations medical curriculum (years 1 and 2).
   1. Free text
2. How many workshops/sessions do you facilitate during the Foundations medical curriculum (years 1 and 2)? (Please choose only one of the following)
   1. 0
   2. 1
   3. 2
   4. 3
   5. 4+
3. What Foundations Course(s) are you part of teaching? (Please choose all that apply)
   1. Foundations of Clinical Sciences (FoCS)
   2. Attacks and Defenses (A&D)
   3. Nutrition, Metabolism, & Gastrointestinal Systems (NMGI)
   4. Neural Sciences
   5. Connections
   6. Cardiovascular, Respiratory & Renal Systems (CRR)
   7. Human Development & Reproductive Health (HDRH)
   8. Convergence
   9. Other: ________
4. Did you interact with the Social Medicine Theme of the Week infographic?
   1. Yes
   2. No
5. How effective are the following interactable elements of the infographic in teaching students about the Social Determinants of Health?

|  | I never engaged with this element | This element is likely to be unhelpful in teaching the Social Determinants of Health | This element is likely to have a minor impact in teaching the Social Determinants of Health | This element is likely to have a major impact in teaching the Social Determinants of Health |
| --- | --- | --- | --- | --- |
| Podcasts |  |  |  |  |
| Videos |  |  |  |  |
| Articles |  |  |  |  |

1. What is one principle or detail in a Social Medicine Theme of the Week infographic that you believe facilitated teaching about social medicine or the Social Determinants of Health well?
   1. Free text
2. Is there anything you would like to see in the infographics that is not consistently included?
   1. Free text
3. Did you feel you were able to successfully incorporate the Social Medicine Theme of the Week into your teaching? (Please choose only one of the following)
   1. Yes
   2. No
4. What prevented you from successfully incorporating the Social Medicine Theme of the Week into your session? (Choose all that apply)
   1. I did not know it existed
   2. I felt like my lecture already included this material
   3. I did not have enough time
   4. I did not have enough training
   5. I did not believe it was important
   6. I could not fit in new material without removing essential material
   7. Other: _______
5. Are there ways you could have been better supported to incorporate the Social Medicine Theme of the Week into your session?
   1. Free text
6. How helpful did you find the Social Medicine Theme of the Week in synthesizing information regarding Social Determinants of Health into coursework? (Please only choose one of the following)
   1. Not helpful at all
   2. A little helpful
   3. Very helpful
7. How did you incorporate teaching about Social Determinants of Health content into your session(s)? (Please choose all that apply)
   1. New slides or pre-reading
   2. Built on existing slide or pre-reading
   3. Mentioned content in class but did not create new material
   4. Revised case presentations
   5. Created discussion questions
   6. Other: _______
8. How challenging did you find it to incorporate teaching about the Social Determinants of Health into your course? (Please only choose one of the following)
   1. Not challenging at all
   2. A little challenging
   3. Very challenging
9. How much additional preparation time did it take to integrate the content into your session? (Please choose only one of the following)
   1. <30 minutes
   2. 30-60 minutes
   3. 60 minutes+
10. Were there any challenges that you faced in developing and delivering this content? (Please choose all that apply)
    1. I did not have prior experience with this topic
    2. I did not feel like I had adequate training in developing or delivering this content
    3. I did not know where to find credible sources
    4. I was unable to answer students’ questions
    5. I did not know how to make the material fit into the academic learning objectives
    6. I was worried about saying something offensive
    7. I did not know how to mediate conflict surrounding this topic
    8. I did not experience any challenges
    9. Other: ___
11. Were there any advantages in developing and delivering this content? (Please choose all that apply)
    1. Students were more engaged with the material
    2. I feel it made the material easier to remember and understand
    3. I learned something new in developing this content
    4. It was personally fulfilling to develop the material
    5. I did not experience any positives
    6. Other: _____
12. Please describe additional resources or supports that would have helped you better integrate the Social Determinants of Health into your teaching sessions.
    1. Free text
13. Would you be interested in getting contacted by a Social Justice Coalition representative to integrate Social Determinants of Health or the Social Medicine Theme of the Week into future teaching sessions?
    1. Yes
    2. No
